# AI analytics can be used as imaging biomarkers for predicting invasive upgrade of ductal carcinoma in situ

**DOI:** 10.1186/s13244-024-01673-0

**Published:** 2024-04-05

**Authors:** Jiyoung Yoon, Juyeon Yang, Hye Sun Lee, Min Jung Kim, Vivian Youngjean Park, Miribi Rho, Jung Hyun Yoon

**Affiliations:** 1 Department of Radiology, Severance Hospital, Research Institute of Radiological Science, Yonsei University College of Medicine, 50-1 Yonsei-ro Seodaemun-gu, Seoul, 03722 South Korea; 2 Biostatistics Collaboration Unit, Yonsei University College of Medicine, 50-1 Yonsei-ro Seodaemun-gu, Seoul, 03722 South Korea

**Keywords:** Artificial intelligence, Breast, Carcinoma (Intraductal noninfiltrating), Image-guided biopsy, Mammography

## Abstract

**Objectives:**

To evaluate whether the quantitative abnormality scores provided by artificial intelligence (AI)-based computer-aided detection/diagnosis (CAD) for mammography interpretation can be used to predict invasive upgrade in ductal carcinoma in situ (DCIS) diagnosed on percutaneous biopsy.

**Methods:**

Four hundred forty DCIS in 420 women (mean age, 52.8 years) diagnosed via percutaneous biopsy from January 2015 to December 2019 were included. Mammographic characteristics were assessed based on imaging features (mammographically occult, mass/asymmetry/distortion, calcifications only, and combined mass/asymmetry/distortion with calcifications) and BI-RADS assessments. Routine pre-biopsy 4-view digital mammograms were analyzed using AI-CAD to obtain abnormality scores (AI-CAD score, ranging 0–100%). Multivariable logistic regression was performed to identify independent predictive mammographic variables after adjusting for clinicopathological variables. A subgroup analysis was performed with mammographically detected DCIS.

**Results:**

Of the 440 DCIS, 117 (26.6%) were upgraded to invasive cancer. Three hundred forty-one (77.5%) DCIS were detected on mammography. The multivariable analysis showed that combined features (odds ratio (OR): 2.225, *p* = 0.033), BI-RADS 4c or 5 assessments (OR: 2.473, *p* = 0.023 and OR: 5.190, *p* < 0.001, respectively), higher AI-CAD score (OR: 1.009, *p* = 0.007), AI-CAD score ≥ 50% (OR: 1.960, *p* = 0.017), and AI-CAD score ≥ 75% (OR: 2.306, *p* = 0.009) were independent predictors of invasive upgrade. In mammographically detected DCIS, combined features (OR: 2.194, *p* = 0.035), and higher AI-CAD score (OR: 1.008, *p* = 0.047) were significant predictors of invasive upgrade.

**Conclusion:**

The AI-CAD score was an independent predictor of invasive upgrade for DCIS. Higher AI-CAD scores, especially in the highest quartile of ≥ 75%, can be used as an objective imaging biomarker to predict invasive upgrade in DCIS diagnosed with percutaneous biopsy.

**Critical relevance statement:**

Noninvasive imaging features including the quantitative results of AI-CAD for mammography interpretation were independent predictors of invasive upgrade in lesions initially diagnosed as ductal carcinoma in situ via percutaneous biopsy and therefore may help decide the direction of surgery before treatment.

**Key points:**

• Predicting ductal carcinoma in situ upgrade is important, yet there is a lack of conclusive non-invasive biomarkers.

• AI-CAD scores—raw numbers, ≥ 50%, and ≥ 75%—predicted ductal carcinoma in situ upgrade independently.

• Quantitative AI-CAD results may help predict ductal carcinoma in situ upgrade and guide patient management.

**Graphical Abstract:**

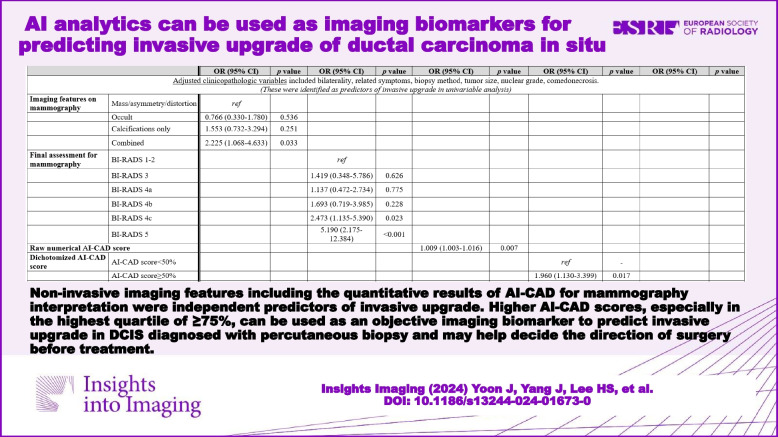

**Supplementary Information:**

The online version contains supplementary material available at 10.1186/s13244-024-01673-0.

## Introduction

Ductal carcinoma in situ (DCIS) is a noninvasive neoplasm of the breast that also displays an unpredictable risk for developing into invasive cancer [1]. As breast cancer screening has become widely available through routine imaging including mammography, the incidence of DCIS has gradually increased to account for approximately one-quarter of new breast cancer diagnoses [2]. While pure DCIS shows a very good prognosis, approximately 14–43% of cases are eventually upgraded to invasive cancer after surgery [3–5], due to the inherent heterogeneous histopathologic features of breast cancer [6]. Therefore, patients with DCIS are treated the same as those with invasive cancer and undergo surgery, radiation therapy, and hormonal therapy [7].

Ongoing trials are investigating active monitoring as an alternative to current standard cancer treatment for low-grade DCIS, which is typically associated with favorable prognosis and often manifests as calcifications on mammography [8–10]. However, studies suggest that candidates presumed to have low-risk DCIS who undergo active monitoring will still face a 5–12% risk of invasive upgrade [11–14]. Currently, there are no factors that reliably predict invasive upgrade after surgery or invasive progression during surveillance.

In an attempt to identify DCIS candidates for less aggressive treatment including active monitoring, previous studies have incorporated clinical or imaging variables to predict invasive upgrade of DCIS after surgery [15–18]. In these studies, the subjective image interpretation of radiologists was used for analysis, limiting the value of the interpretation in terms of data reproducibility or consistency. There has also been an attempt to predict invasive upgrades of DCIS using a machine-learning model based on mammographic radiomic features. However, the complexity of extracting and utilizing radiomic features in actual clinical settings is another challenge in addition to clinically incorporating the model [19]. Recently, artificial intelligence (AI)-based computer-aided detection/diagnosis (CAD) algorithms were approved for commercial use in mammography interpretation [20], and the software programs provide quantitative numeric data for abnormalities detected on mammography images. The quantitative analytic data obtained from AI-CAD may be consistent and objective imaging features compared to radiologists’ interpretations. However, to the best of our knowledge, there are no studies evaluating the utilization of abnormality scores provided by AI-CAD systems for mammographic interpretation to predict DCIS upgrade.

Thus, the purpose of this study was to evaluate whether the quantitative abnormality scores provided by AI-CAD for mammography interpretation can be used to predict invasive upgrade in DCIS diagnosed with percutaneous biopsy.

## Methods

The institutional review board (IRB) of Severance Hospital, Yonsei University (IRB approval No: 4-2022-0519) approved this retrospective study and waived the requirement for informed consent based on its study design.

### Study population

**Fig. 1:**
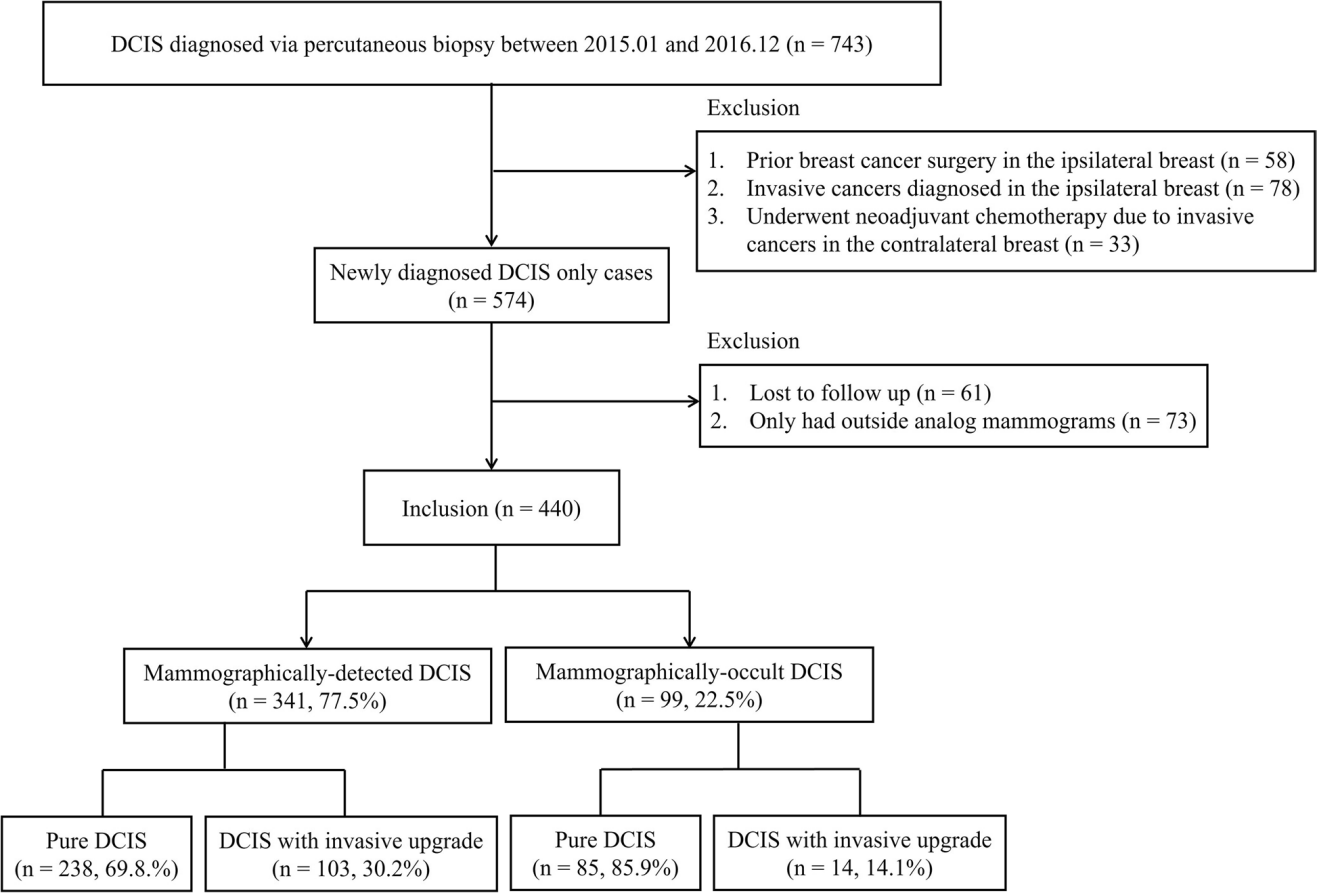
Flowchart of patient selection

We searched our institutional database for women who were diagnosed with DCIS via percutaneous biopsy, including core needle biopsy and imaging-guided vacuum-assisted biopsy (VAB). From January 2015 to December 2019, 743 DCIS were diagnosed in 717 women via image-guided percutaneous biopsy. The exclusion criteria were as follows: (1) women who were surgically treated for breast cancer in the ipsilateral breast (*n* = 58), (2) women who had invasive cancers diagnosed in the ipsilateral breast (*n* = 78), (3) women who underwent neoadjuvant chemotherapy due to invasive cancers in the contralateral breast (*n* = 33), (4) women who were lost to follow-up after DCIS diagnosis (*n* = 61), and (5) women who only had analog mammograms from outside hospitals that were inadequate for AI-CAD analysis (*n* = 73) (Fig. 1).

The clinical characteristics of patients including age, family history of breast cancer, personal history of breast cancer, presence of bilateral breast cancer, presence of related symptoms such as palpability or nipple discharge, method of percutaneous biopsy, and tumor size were extracted from our electronic medical record (EMR) system.

### Mammography examinations and interpretation

One of two dedicated digital mammography units was used for the mammography examinations (Senographe DS, GE Medical Systems; Lorad Selenia, Hologic). Standard mediolateral oblique (MLO) and craniocaudal (CC) mammograms and magnification views with 90° lateral and craniocaudal projections, if required, were obtained for all patients.

One board-certified, breast-dedicated radiologist with 14 years of experience in breast imaging (J.H.Y.) retrospectively reviewed the baseline mammograms that were collected routinely before biopsy. Mammographic features of abnormalities that correlated to the biopsy-proven DCIS were categorized into the following four categories: (1) mammographically occult (DCIS detected on supplemental ultrasound (US)), (2) mass/asymmetry/distortion, (3) calcifications only, and (4) combined mass/asymmetry/distortion with calcifications (referred to as “combined features”). Final assessments according to the American College of Radiology Breast Imaging Reporting And Data System (ACR BI-RADS) [21] were also determined by the radiologist during the retrospective review. The radiologist was blinded to the final surgical diagnosis.

### Mammography analysis using AI-CAD

A commercially available AI-CAD algorithm (Lunit INSIGHT for Mammography, version 1.1.4.3, Lunit Inc., Seoul, Korea) that was previously validated through a multinational study [22] was used for analyzing mammograms. The algorithm, based on the ResNet-34, a popular deep convolutional neural network (CNN) architecture, was trained using 31,604 cancer-positive mammograms and 19,625 benign mammograms with pixel-label labels indicating lesion locations annotated by 12 breast-dedicated radiologists. The algorithm provides region of interest (ROI) marks for abnormalities on mammograms while providing corresponding abnormality scores (referred to as AI-CAD scores, ranging 0–100%) per view.

In this study, we employed a three-pronged approach for AI-CAD scores: (1) numerical AI-CAD score provided in raw numbers (ranging from 0 to 100%); (2) AI-CAD scores dichotomized into < 50% and ≥ 50%; and (3) graded AI-CAD score of < 25%, 25–50%, 50–75%, and ≥ 75%.

### Histopathology at percutaneous biopsy

Information regarding nuclear grade (low, intermediate, or high grade) and presence of comedonecrosis was collected from the pathology reports from percutaneous biopsy. Tumors on percutaneous biopsy specimens were histologically classified using the World Health Organization criteria [23].

### Statistical analysis

Ground truth in terms of pure DCIS or invasive cancer was confirmed after surgery. The Shapiro-Wilk test and Kolmogorov-Smirnov test were performed to test for normality for age, tumor size, and AI-CAD scores. As the normality assumption was not satisfied, the median values for these factors were calculated and compared. The Mann-Whitney *U* test was used to compare clinicopathological variables between pure DCIS and DCIS with invasive upgrade. Mammographic variables such as imaging features on mammography, ACR-BI-RADS final assessment, and median AI-CAD score were also compared between pure DCIS and DCIS with invasive upgrade using the Mann-Whitney *U* test and Fisher’s exact test.

Univariable logistic regression analysis using clinicopathological variables and mammographic variables was performed to assess predicting factors for invasive upgrade in DCIS. Subsequently, multivariable logistic regression analysis was performed to identify independent predictive mammographic variables after adjusting for clinicopathological variables. Variables with *p* values less than 0.05 in the univariable logistic regression analysis were included for multivariable logistic regression analysis. The predictability of the multivariable models was evaluated with the area under the receiver operating characteristics curve (AUROC). A subgroup analysis was conducted specifically on DCIS detected on mammography, referred to as “mammographically detected DCIS.” This analysis excluded cases that were mammographically occult, to simulate situations where supplemental screening with imaging modalities other than mammography is not common.

All statistical analyses were performed using SAS (version 9.4, SAS Inc.). *p-*values less than 0.05 were considered statistically significant.

## Results

In total, 440 DCIS diagnosed via percutaneous biopsy in 420 women were included in this study. Three hundred thirty-three were diagnosed using US-guided core needle biopsy, and 107 through VAB. Twenty women were diagnosed with DCIS in both breasts, and these lesions were all included in our analysis as AI-CAD for mammography provides a per-breast analysis. Within the VAB group, 37 patients were diagnosed via stereotactic biopsy (mammogram-guided VAB), while 70 were diagnosed using US-guided VAB. None of the enrolled patients underwent biopsy under MRI guidance. Of the 440 lesions that were diagnosed as DCIS by percutaneous biopsy, 117 (26.6%) lesions were upgraded to invasive disease after surgery (Fig. 1). Breast US is commonly employed as a supplemental imaging tool for screening breast cancer in Korea, and a significant proportion of our population (22.5%, 99 of 440) had DCIS detected on supplemental screening US. In the subgroup analysis of mammographically detected DCIS (*n* = 341), 103 (30.2%) lesions were upgraded to invasive disease after surgery. The mean patient age was 52.8 years (range, 28–85 years).

Table 1 compares clinicopathologic factors between pure DCIS and DCIS with invasive upgrade. Among clinicopathological factors, significantly higher rates of unilateral DCIS at diagnosis, presence of symptoms, core needle biopsy as the biopsy method, larger tumor size, intermediate or high nuclear grades, and the presence of comedonecrosis were observed in DCIS with invasive upgrade (all *p* < 0.05, respectively). Similar findings were seen in the subgroup of mammographically detected DCIS, except that the presence of bilateral DCIS and comedonecrosis did not show significant differences between pure DCIS and DCIS with invasive upgrade (*p* = 0.138 and 0.086, respectively).

**Table 1:** Comparison of clinicopathological factors between pure DCIS and DCIS with invasive upgrade

	**Total DCIS (*n* = 440)**			**Mammographically detected DCIS (*n* = 341)**		
	**Pure DCIS (*n* = 323)**	**DCIS with invasive upgrade (*n* = 117)**	***p* value**	**Pure DCIS (*n* = 238)**	**DCIS with invasive upgrade (*n* = 103)**	***p* value**
***Clinical factors***						
Age (years, median (Q1, Q3))	52.0 (45.0, 61.0)	50.0 (46.0, 59.0)	0.298	53.0 (46.0, 61.0)	50.0 (46.0, 60.0)	0.339
Family history of breast cancer			0.410			0.483
No	296 (91.6)	110 (94.0)		219 (92.0)	97 (94.2)	
Yes	27 (8.4)	7 (6.0)		19 (8.0)	6 (5.8)	
Personal history of breast cancer			> 0.999			> 0.999
No	310 (96.0)	113 (96.6)		234 (98.3)	102 (99.0)	
Yes	13 (4.0)	4 (3.4)		4 (1.7)	1 (1.0)	
Bilateral DCIS			0.006			0.138
No	263 (81.4)	108 (92.3)		212 (89.1)	97 (94.2)	
Yes	60 (18.6)	9 (7.7)		26 (10.9)	6 (5.8)	
Related symptoms			0.010			0.018
Asymptomatic	261 (80.8)	81 (69.2)		186 (78.2)	68 (66.0)	
Present	62 (19.2)	36 (30.8)		52 (21.8)	35 (34.0)	
Biopsy method			0.009			0.003
Core needle biopsy	234 (72.5)	99 (84.6)		159 (66.8)	85 (82.5)	
Vacuum-assisted biopsy	89 (27.5)	18 (15.4)		79 (33.2)	18 (17.5)	
Tumor size (mm, median (Q1, Q3))	14.0 (8.0, 23.0)	20.00 (12.0, 30.0)	0.002	17.0 (10.0, 26.0)	21.0 (13.0, 30.0)	0.012
***Pathologic factors***						
Nuclear grade			0.001			0.006
Low	134 (41.5)	28 (23.9)		84 (35.3)	21 (20.4)	
Intermediate	158 (48.9)	67 (57.3)		126 (52.9)	60 (58.3)	
High	31 (9.6)	22 (18.8)		28 (11.8)	22 (21.4)	
Comedonecrosis			0.012			0.086
No	151 (46.8)	39 (33.3)		90 (37.8)	29 (28.2)	
Yes	172 (53.2)	78 (66.7)		148 (62.2)	74 (71.8)	

Percentages are in parentheses. *DCIS* Ductal carcinoma in situ, *Q1* First quartile, *Q3* Third quartile

Table 2 compares mammographic factors between pure DCIS and DCIS with invasive upgrade. Regarding mammographic factors, significantly higher rates of combined features, BI-RADS 4C and 5 assessments, higher median AI-CAD scores, and higher rates of AI-CAD scores ≥ 50% and ≥ 75% were seen in DCIS with invasive upgrade (all *p* < 0.001, respectively) for the total DCIS and mammographically detected DCIS.

**Table 2:** Comparison of mammographic factors between pure DCIS and DCIS with invasive upgrade

	**Total DCIS (*n* = 440)**			**Mammographically detected DCIS (*n* = 341)**		
	**Pure DCIS (*n* = 323)**	**DCIS with invasive upgrade (*n* = 117)**	***p* value**	**Pure DCIS (*n* = 238)**	**DCIS with invasive upgrade (*n* = 103)**	***p* value**
**Imaging features on mammography**			< 0.001			0.007
Occult	85 (26.3)	14 (21.0)		-	-	
Calcifications only	123 (38.1)	47 (40.2)		123 (51.7)	47 (45.6)	
Mass/asymmetry/distortion	58 (18.0)	15 (12.8)		58 (24.4)	15 (14.6)	
Combined	57 (17.7)	41 (35.0)		57 (23.9)	41 (39.8)	
**Final assessment on mammography**			< 0.001			< 0.001
BI-RADS 1–2	85 (26.3)	14 (12.0)		-	-	
BI-RADS 3	12 (3.7)	3 (2.5)		12 (5.0)	3 (2.9)	
BI-RADS 4a	65 (20.1)	12 (10.3)		65 (27.3)	12 (11.7)	
BI-RADS 4b	55 (17.0)	16 (13.7)		55 (23.1)	16 (15.5)	
BI-RADS 4c	74 (22.9)	33 (28.2)		74 (31.1)	33 (32.0)	
BI-RADS 5	32 (9.9)	39 (33.3)		32 (13.4)	39 (37.9)	
**Raw numerical AI-CAD score (%, median [Q1,Q3])**	56.9 (5.9, 97.3)	96.6 (44.8, 99.5)	< 0.001	88.9 (33.1, 98.7)	98.1 (83.0, 99.6)	< 0.001
**Dichotomized AI-CAD score**			< 0.001			0.002
AI-CAD score < 50%	155 (48.0)	30 (25.6)		76 (31.3)	16 (15.5)	
AI-CAD score ≥ 50%	168 (52.0)	87 (74.4)		167 (68.7)	87 (84.5)	
**Graded AI-CAD score**			< 0.001			0.016
AI-CAD score < 25%	123 (38.1)	22 (18.8)		47 (19.7)	9 (8.7)	
AI-CAD score 25–50%	32 (9.9)	8 (6.8)		24 (10.1)	7 (6.8)	
AI-CAD score 50–75%	27 (8.4)	8 (6.8)		26 (10.9)	8 (7.8)	
AI-CAD score ≥ 75%	141 (43.7)	79 (67.5)		141 (59.2)	79 (76.7)	

Percentages are in parentheses. *DCIS* Ductal carcinoma in situ, *Q1* First quartile, *Q3* Third quartile, *BI-RADS* Breast Imaging Reporting And Data System, *AI-CAD* Artificial intelligence-based computer-aided detection/diagnosis

### Prediction of upgrade to invasive carcinoma

For the total 440 DCIS, univariable logistic regression analysis demonstrated that clinicopathological variables including unilateral DCIS at diagnosis, presence of related symptoms, core needle biopsy as the biopsy method, larger tumor size, intermediate or high nuclear grade, and presence of comedonecrosis were predictors of invasive upgrade (all *p* < 0.05, respectively). In the subgroup of mammographically detected lesions (*n* = 341), presence of related symptoms, biopsy method, and nuclear grade were significantly associated with invasive upgrade (Additional file 1: Table S1).

For mammographic variables, combined features, higher BI-RADS assessments, and higher AI-CAD scores, either as raw numbers or in grades, were significantly associated with invasive upgrade (all *p* < 0.05, respectively) in the total DCIS and mammographically detected DCIS (Additional file 1: Table S1).

Table 3 summarizes the multivariable logistic regression analysis results in the total DCIS group. After adjusting for clinicopathological variables, mammographic variables of combined features (odds ratio (OR): 2.225, 95% confidence interval (CI): 1.068–4.633, *p* = 0.033), BI-RADS 4c and 5 assessments (OR: 2.473, 95% CI: 1.135–5.390, *p* = 0.023 and OR: 5.190, 95% CI: 2.175–12.384, *p* < 0.001, respectively), higher AI-CAD score (1.009, 95% CI: 1.003–1.016, *p* = 0.007), AI-CAD score ≥ 50% (OR: 1.960, 95% CI: 1.130–3.399, *p* = 0.017), and AI-CAD score ≥ 75% (OR: 2.306, 95% CI: 1.233–4.313, *p* = 0.009) were all independent predictors of invasive upgrade. Multivariable logistic regression analysis in mammographically detected DCIS showed that combined features (OR: 2.194, 95% CI: 1.057–4.557, *p* = 0.035) and higher AI-CAD score (OR: 1.008, 95% CI: 1.000–1.017, *p* = 0.047) were independent predictors of invasive upgrade (Table 4). However, the final BI-RADS assessment, AI-CAD score ≥ 50%, and AI-CAD score ≥ 75% were not independent predictors for invasive upgrade in mammographically detected DCIS.

**Table 3:** Multivariable analysis for predictors of invasive upgrade in the total 440 DCIS patients

**Variables**		**Clinicopathologic variables + mammographic features**		**Clinicopathologic variables + BI-RADS assessments**		**Clinicopathologic variables + raw numerical AI-CAD scores**		**Clinicopathologic features + dichotomized AI-CAD score**		**Clinicopathologic features + graded AI-CAD score**	
		**OR (95% CI)**	***p* value**	**OR (95% CI)**	***p* value**	**OR (95% CI)**	***p* value**	**OR (95% CI)**	***p* value**	**OR (95% CI)**	***p* value**
**Bilateral breast cancer**	No	*ref*		*ref*		*ref*		*ref*		*ref*	
	Yes	0.527 (0.240–1.157)	0.110	0.558 (0.252–1.235)	0.150	0.533 (0.243–1.167)	0.115	0.508 (0.233–1.107)	0.0883	0.530 (0.242–1.162)	0.113
**Related symptoms**	No	*ref*		*ref*		*ref*		*ref*		*ref*	
	Yes	1.290 (0.731–2.278)	0.380	1.222 (0.695–2.148)	0.486	1.348 (0.780–2.331)	0.285	1.331 (0.770–2.300)	0.3054	1.356 (0.782–2.351)	0.279
**Biopsy method**	Core needle biopsy	*ref*		*ref*		*ref*		*ref*		*ref*	
	Vacuum-assisted biopsy	0.380 (0.203–0.712)	0.003	0.466 (0.249–0.874)	0.017	0.390 (0.212–0.716)	0.002	0.384 (0.209–0.705)	0.002	0.380 (0.206–0.698)	0.002
**Tumor size**		1.004 (0.988–1.021)	0.610	0.997 (0.980–1.014)	0.720	1.002 (0.986–1.018)	0.827	1.004 (0.987–1.020)	0.667	1.002 (0.986–1.018)	0.819
**Nuclear grade**	Low	*ref*		*ref*		*ref*		*ref*		*ref*	
	Intermediate	1.973 (1.046–3.722)	0.036	1.830 (0.964–3.475)	0.065	1.779 (0.938–3.373)	0.078	1.839 (0.971–3.483)	0.062	1.827 (0.963–3.465)	0.065
	High	2.624 (1.130–6.096)	0.025	2.124 (0.893–5.048)	0.088	2.432 (1.045–5.663)	0.039	2.539 (1.094–5.893)	0.030	2.455 (1.055–5.714)	0.037
**Comedonecrosis**	No	*ref*		*ref*		*ref*		*ref*		*ref*	
	Yes	0.920 (0.510–1.660)	0.78	0.867 (0.473–1.587)	0.643	0.950 (0.527–1.712)	0.863	0.969 (0.538–1.745)	0.915	0.945 (0.524–1.705)	0.850
**Imaging features on mammography**	Mass/asymmetry/distortion	*ref*									
	Occult	0.766 (0.330–1.780)	0.536								
	Calcifications only	1.553 (0.732–3.294)	0.251								
	Combined	2.225 (1.068–4.633)	0.033								
**Final assessment for mammography**	BI-RADS 1–2			*ref*							
	BI-RADS 3			1.419 (0.348–5.786)	0.626						
	BI-RADS 4a			1.137 (0.472–2.734)	0.775						
	BI-RADS 4b			1.693 (0.719–3.985)	0.228						
	BI-RADS 4c			2.473 (1.135–5.390)	0.023						
	BI-RADS 5			5.190 (2.175–12.384)	< 0.001						
**Raw numerical** A**I-CAD score**						1.009 (1.003–1.016)	0.007				
**Dichotomized AI-CAD score**	AI-CAD score < 50%							*ref*			
	AI-CAD score ≥ 50%							1.960 (1.130–3.399)	0.017		
**Graded AI-CAD score**	AI-CAD score < 25%									*ref*	
	AI-CAD score 25–50%									1.234 (0.489–3.112)	0.657
	AI-CAD score 50–75%									1.260 (0.487–3.264)	0.634
	AI-CAD score ≥ 75%									2.306 (1.233–4.313)	0.009
**AUC (95% CI)**		0.708 (0.655–0.761)		0.724 (0.671–0.777)		0.702 (0.648–0.755)		0.699 (0.645–0.753)		0.703 (0.649–0.757)	

*DCIS* Ductal carcinoma in situ, *OR* Odds ratio, *95% CI* 95% confidence interval, *ref* Reference, *BI-RADS* Breast Imaging Reporting And Data System, *AI-CAD* AI-based computer-aided detection/diagnosis, *AUC* Area under curve

**Table 4:** Multivariable analysis for predictors of invasive upgrade in the 341 mammographically detected DCIS patients

**Variables**		**Clinicopathologic features + mammographic features**		**Clinicopathologic features + BI-RADS assessments**		**Clinicopathologic features + AI-CAD scores**		**Clinicopathologic features + dichotomized AI-CAD score**		**Clinicopathologic features + graded AI-CAD score**	
		**OR (95% CI)**	***p* value**	**OR (95% CI)**	***p* value**	**OR (95% CI)**	***p* value**	**OR (95% CI)**	***p* value**	**OR (95% CI)**	***p* value**
**Related symptoms**	No	*ref*		*ref*		*ref*		*ref*		*ref*	
	Yes	1.518 (0.860–2.681)	0.150	1.317 (0.746–2.323)	0.343	1.534 (0.887–2.652)	0.126	1.533 (0.887–2.652)	0.126	1.537 (0.886–2.665)	0.126
**Biopsy method**	Core needle biopsy	*ref*		*ref*		*ref*		*ref*		*ref*	
	Vacuum-assisted biopsy	0.419 (0.223–0.787)	0.007	0.495 (0.264–0.929)	0.029	0.417 (0.227–0.768)	0.005	0.411 (0.223–0.756)	0.004	0.406 (0.220–0.748)	0.004
**Nuclear grade**	Low	*ref*		*ref*		*ref*		*ref*		*ref*	
	Intermediate	2.115 (1.138–3.931)	0.018	1.773 (0.953–3.298)	0.070	1.878 (1.003–3.515)	0.049	1.990 (1.071–3.697)	0.029	1.925 (1.033–3.587)	0.039
	High	3.028 (1.370–6.690)	0.006	2.148 (0.949–4.862)	0.067	2.709 (1.223–6.002)	0.014	2.915 (1.330–6.388)	0.008	2.725 (1.236–6.012)	0.013
**Imaging features on mammography**	Mass/asymmetry/distortion	*ref*									
	Calcifications only	1.519 (0.716–3.223)	0.276								
	Combined	2.194 (1.057–4.557)	0.035								
**Final assessment for mammography**	BI-RADS 3			*ref*							
	BI-RADS 4a			0.816 (0.196–3.399)	0.780						
	BI-RADS 4b			1.190 (0.287–4.932)	0.811						
	BI-RADS 4c			1.660 (0.423–6.511)	0.467						
	BI-RADS 5			3.507 (0.861–14.280)	0.080						
**Raw numerical AI-CAD score**						1.008 (1.000–1.017)	0.047				
**Dichotomized AI-CAD score**	AI-CAD score < 50%							*ref*			
	AI-CAD socre ≥ 50%							1.735 (0.904–3.330)	0.098		
**Graded AI-CAD score**	AI-CAD score < 25%									*ref*	
	AI-CAD score 25–50%									1.371 (0.443–4.249)	0.584
	AI-CAD score 50–75%									1.191 (0.394–3.601)	0.757
	AI-CAD score ≥ 75%									2.145 (0.944–4.873)	0.069
**AUC (95% CI)**		0.691 (0.630–0.751)		0.712 (0.653–0.772)		0.688 (0.627–0.749)		0.677 (0.615–0.738)		0.683 (0.623–0.744)	

*DCIS* Ductal carcinoma in situ, *OR* Odds ratio, *95% CI* 95% confidence interval, *ref* Reference, *BI-RADS* Breast Imaging Reporting And Data System, *AI-CAD* AI-based computer-aided detection/diagnosis, *AUC* Area under curve

## Discussion

In this study, we investigated the clinicopathologic and mammographic factors associated with DCIS upgrade to invasive cancer after surgery. Clinicopathologic factors such as biopsy method and nuclear grade on biopsy were significant predictors of invasive upgrade. Among mammographic factors, combined features, BI-RADS 4c and 5 assessments, and AI-CAD scores—raw numbers, ≥ 50%, and ≥ 75%—were independent predictors of invasive upgrade. In the subgroup analysis of mammographically detected DCIS, the AI-CAD scores in raw numbers remained an independent predictor for invasive upgrade.

Our results showed that DCIS presenting as combined mass/asymmetry/distortion with calcifications had a significantly higher likelihood of being upgraded to invasive cancer. In the multivariable logistic regression analysis, OR was 2.225 and 2.194 for the total 440 DCIS and the mammographically detected DCIS, respectively. The association between DCIS presenting as soft tissue lesions combined with calcifications and invasive upgrade has been previously reported [3, 24]. In the meta-analysis by Brennan et al. [3], DCIS appearing as a mass with calcifications on mammography was significantly associated with invasive upgrade with an OR of 1.83. A higher level of suspicion for breast cancer indicated by a higher BI-RADS final assessment made by a radiologist was also associated with an increased rate of DCIS with invasive upgrade, as in prior studies [3, 25]. When suspicious mass/asymmetry/distortion is accompanied by suspicious calcifications, final assessments are commonly elevated. Therefore, the association between combined features and invasive upgrade of DCIS aligns with the association between higher BI-RADS assessment and DCIS upgrade.

**Fig. 2:**
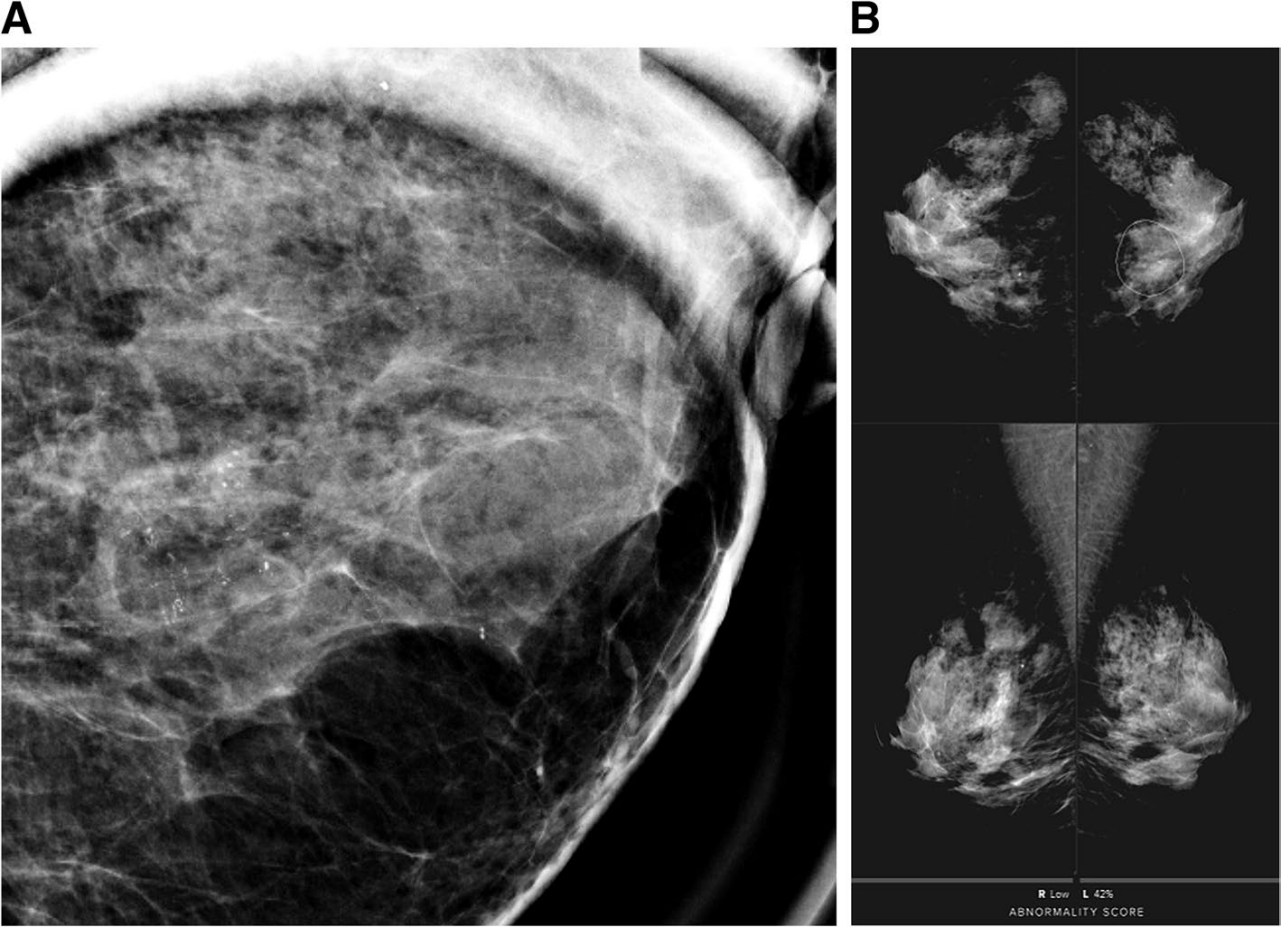
A 49-year-old female patient with pure ductal carcinoma in situ. The patient was referred to our hospital due to a screening-detected calcification in the left lower medial breast. The calcification was assessed as BI-RADS category 4B by the breast radiologist (**A**) and the AI-CAD score was 42% (**B**). A stereotactic biopsy was performed, confirming ductal carcinoma in situ. Following partial mastectomy, the final pathology revealed pure ductal carcinoma in situ with intermediate nuclear grade and the presence of comedo necrosis

**Fig. 3:**
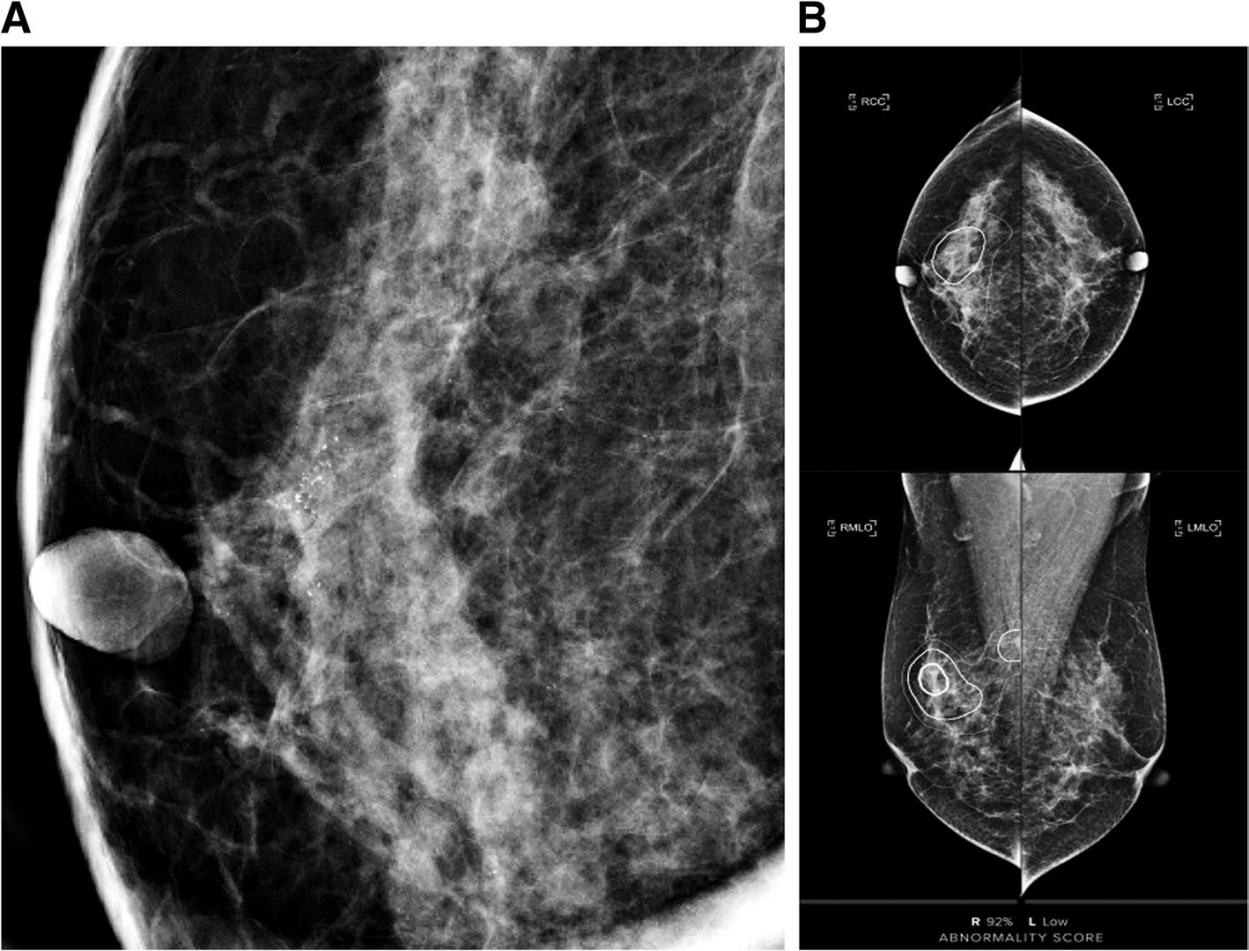
A 37-year-old female patient with ductal carcinoma in situ with invasive upgrade. The patient was referred to our hospital due to a screening-detected calcification in the right upper outer and upper central breast. The calcification was assessed as BI-RADS category 4B by the breast radiologist (**A**) and the AI-CAD score was 92% (**B**). A stereotactic biopsy was performed, confirming ductal carcinoma in situ. However, after nipple-sparing mastectomy, the final pathology revealed invasive ductal carcinoma. No metastatic lymph nodes were found on sentinel lymph node biopsy

Having radiologists evaluate abnormal findings on mammography possesses inherent limitations due to subjectivity and low reproducibility [26]. Notable disagreements have been observed among radiologists with various levels of experience when using BI-RADS descriptors and assessments for mammographic interpretation [27, 28]. In this aspect, image analysis data provided by AI-CAD can be used not only to assist image interpretation but also as objective imaging biomarkers. In this aspect, we evaluated the analysis data of a commercially available AI-CAD system designed for mammographic interpretation to see if the information could be used as biomarkers to predict invasive upgrade in DCIS diagnosed by percutaneous biopsy. Our results showed that the AI-CAD score could potentially predict invasive upgrade in DCIS: higher AI-CAD score (OR 1.009), AI-CAD score ≥ 50% (OR 1.960), and AI-CAD score ≥ 75% (OR 2.306) were independent predictors of invasive upgrade in the total DCIS, and a higher AI-CAD score was an independent predictor of invasive upgrade (OR 1.008) in mammographically detected DCIS. Based on our results, DCIS with higher AI-CAD scores, especially in the highest quartile of ≥ 75%, can be considered to have invasive components (Figs. 2 and 3).

When combining clinicopathologic variables with AI-CAD scores to construct a predictive model for invasive upgrade, we achieved acceptable diagnostic performance with an AUROC of 0.699–0.703 for total DCIS (Table 3) and an AUROC of 0.677–0.688 for mammographically detected DCIS (Table 4). These results are comparable to the AUROCs of 0.71 (95% CI, 0.67–0.75) and 0.70 (95% CI, 0.68–0.73) reported in prior studies, where algorithms predicting invasive upgrade were developed by applying CNN to the mammography images of biopsy-proven DCIS patients [29, 30]. Notably, our study utilized a commercially available AI algorithm designed for breast cancer detection, rather than an AI algorithm specifically trained to predict invasive upgrade. In this context, comparable outcomes can be considered highly promising, as this achievement suggests that pre-existing AI technologies can be used to predict invasive upgrade.

Predicting DCIS upgrade before surgery is crucial for surgical decision-making, as the overall incidence of axillary lymph node metastasis is around 5% but rises to 20% for preoperatively underestimated lesions [31–33]. This highlights the need for performing sentinel biopsies on patients with a strong preoperative upgrade prediction to prevent additional completion surgery. Moreover, given the current trend towards less aggressive treatment for low-risk DCIS including the omission of surgery [8–10, 34], accurately identifying DCIS with invasive components becomes vital in selecting the right patients for active monitoring. Our results show that in addition to clinicopathological predictors such as biopsy method or nuclear grades, imaging features either evaluated by radiologists or AI-CAD can be used as biomarkers for predicting invasive upgrade. We look forward to future studies that can validate our results.

Interestingly, the imaging predictors that significantly predicted invasive upgrade in total DCIS—BI-RADS assessment by the radiologist, AI-CAD score ≥ 50%, and ≥ 75%—lost their significance within the mammographically detected DCIS subgroup. Approximately 78.6% (268 of 341) of mammographically detected DCIS in our study presented with calcifications, and this may have attributed to this result. Calcifications representative of DCIS have a broad range of imaging features from round/amorphous, benign-appearing calcifications to fine pleomorphic/fine linear branching suspicious calcifications where the BI-RADS assessments may have been evenly distributed among the BI-RADS 4 subgroup. Radiologists encounter difficulties assessing abnormalities with ambiguous, overlapping imaging features, which seems to be the same for AI-CAD as dichotomized or graded AI-CAD scores could not independently predict DCIS upgrade in mammographically detected ones. Nonetheless, raw numerical AI-CAD scores showed significance even in the mammographically detected DCIS, supporting our claim that quantitative AI-CAD assessment has the potential to provide more substantial predictive value compared to radiologists when identifying invasive upgrades within DCIS diagnosed through percutaneous biopsy.

There are several limitations to our study. First, this was a retrospective study performed in a single institution, and therefore, selection bias was inevitable. Also, the women included in this study were all Asian, and most had mammographically dense breasts (88.4% with breasts assessed as parenchymal density grade C or D). Second, mammographic features of DCIS and the BI-RADS final assessments were determined by one experienced breast radiologist. Results may differ when readers with varying levels of experience are involved in mammography interpretation. Third, we only used one commercially available AI-CAD system, and our results cannot be generalized to other AI platforms.

## Conclusion

In conclusion, when applying AI-CAD for mammography, the AI-CAD score was an independent predictor of invasive upgrade in DCIS. Higher AI-CAD scores, especially in the highest quartile of ≥ 75%, can be used as an objective imaging biomarker to predict invasive upgrade in DCIS diagnosed with percutaneous biopsy.

### Supplementary Information

**Supplementary Material 1.** 

## Data Availability

The datasets generated or analyzed during the study are available from the corresponding author on reasonable request.

## Abbreviations

ACR BI-RADS: American College of Radiology Breast Imaging Reporting And Data System
AI: Articifial intelligence
AUROC: Area under the receiver operating characteristics curve
CAD: Computer-aided detection/diagnosis
CI: Confidence interval
CNN: Convolutional neural network
DCIS: Ductal carcinoma in situ
IRB: Instituional review board
OR: Odds ratio
US: Ultrasound
VAB: Vacuum-assisted biopsy
