# Supplementary material for: AI analytics can be used as imaging biomarkers for predicting invasive upgrade of ductal carcinoma in situ
Source: Insights Imaging. 2024 Apr 5;15:100. doi: 10.1186/s13244-024-01673-0 (PMC10997564; doi:10.1186/s13244-024-01673-0)
Supplement: Supplementary file 1 — Supplementary Material 1. [file 13244_2024_1673_MOESM1_ESM.pdf]

AI Analytics Can be Used as Imaging Biomarkers for Predicting Invasive Upgrade of Ductal carcinoma in situ

ELECTRONIC SUPPLEMENTARY MATERIAL

Table S1. Univariable analysis of predictors for invasive upgrade in patients with initial diagnosis of DCIS

		Total DCIS (n=440)		Mammographically detected DCIS (n=341)	
		OR (95% CI)	*p* value	OR (95% CI)	*p* value
Clinical variables					
**Age**		0.989 (0.969-1.009)	0.271	0.989 (0.967-1.011)	0.316
**Family history of breast cancer**	No	*ref*		*ref*	
	Yes	0.698 (0.295-1.648)	0.412	0.713 (0.276-1.841)	0.485
**Personal history of breast cancer**	No	*ref*		*ref*	
	Yes	0.844 (0.270-2.642)	0.771	0.574 (0.063-5.197)	0.622
**Bilateral DCIS**	No	*ref*		*ref*	
	Yes	0.365 (0.175-0.762)	0.007	0.504 (0.201-1.265)	0.145
**Presence of symptoms**	No	*ref*		*ref*	
	Yes	1.871 (1.158-3.026)	0.011	1.841 (1.105-3.068)	0.019
**Biopsy method**	Core needle biopsy	*ref*		*ref*	
	Vacuum-assisted biopsy	0.478 (0.274-0.836)	0.010	0.426 (0.240-0.758)	0.004
**Tumor size**		1.014 (1.000-1.027)	0.048	1.010 (0.995-1.024)	0.182
Pathologic variables					
**Nuclear grade**	Low	*ref*		*ref*	
	Intermediate	2.029 (1.234-3.338)	0.005	1.905 (1.079-3.363)	0.026
	High grade	3.396 (1.718-6.714)	<0.001	3.143 (1.507-6.555)	0.002
**Comedonecrosis**	No	*ref*		*ref*	
	Yes	1.755 (1.128-2.732)	0.013	1.552 (0.938-2.566)	0.087
Mammographic variables					
**Imaging features on mammography**	Mass/asymmetry/distortion	*ref*		*ref*	
	Occult	0.637 (0.286-1.419)	0.270	-	-
	Calcifications only	1.478 (0.764-2.858)	0.246	1.478 (0.764-2.858)	0.246
	Combined	2.781 (1.388-5.574)	0.004	2.781 (1.388-5.574)	0.004
**Final assessment on mammography**	BI-RADS 1-2	*ref*		-	-
	BI-RADS 3	1.518 (0.380-6.068)	0.555	*ref*	
	BI-RADS 4a	1.121 (0.486-2.586)	0.789	0.738 (0.181-	0.673

				3.016)	
	BI-RADS 4b	1.766 (0.799-3.905)	0.160	1.164 (0.292-4.636)	0.830
	BI-RADS 4c	2.707 (1.347-5.444)	0.005	1.784 (0.472-6.745)	0.394
	BI-RADS 5	7.399 (3.553-15.409)	<0.001	4.875 (1.265-18.782)	0.021
**Raw numerical AI-CAD score**		1.013 (1.007-1.019)	<0.001	1.012 (1.005-1.020)	0.002
**Dichotomized AI-CAD score**	AI-CAD score<50%	*ref*		*ref*	
	AI-CAD score≥50%	2.676 (1.674-4.276)	<.001	2.312 (1.267-4.217)	0.006
**Graded AI-CAD score**	AI-CAD score<25%	*ref*		*ref*	
	AI-CAD score 25-50%	1.398 (0.570-3.430)	0.465	1.523 (0.505-4.591)	0.455
	AI-CAD score 50-75%	1.657 (0.667-4.116)	0.277	1.607 (0.553-4.666)	0.383
	AI-CAD score≥75%	3.132 (1.842-5.326)	<.001	2.926 (1.362-6.284)	0.006

Note.—DCIS = ductal carcinoma in situ. OR = odds ratio.95% CI = 95% confidence interval.

ref = reference. BI-RADS = Breast Imaging Reporting And Data System. AI-CAD = AI-based computer-aided detection/diagnosis
